# Comparison of muscle quality and functional capacity between Japanese and Brazilian older individuals

**DOI:** 10.1371/journal.pone.0243589

**Published:** 2020-12-16

**Authors:** Hiroshi Akima, Akito Yoshiko, Régis Radaelli, Madoka Ogawa, Kaori Shimizu, Aya Tomita, Hisashi Maeda, Rodrigo Neske, Juliana Teodoro, Kazuhiro Maeda, Noriko Tanaka, Ronei Pinto

**Affiliations:** 1 Research Center of Health, Physical Fitness & Sports, Nagoya University, Nagoya, Aichi, Japan; 2 Graduate School of Education & Human Development, Nagoya University, Nagoya, Aichi, Japan; 3 School of International Liberal Studies, Chukyo University, Toyota, Aichi, Japan; 4 Exercise Research Laboratory (LAPEX), Federal University of Rio Grande do Sul (UFRGS), Port Alegre, RS, Brazil; 5 Faculty of Sport Science, Nippon Sport Science University, Setagaya, Tokyo, Japan; 6 Graduate School of Medicine, Nagoya University, Nagoya, Aichi, Japan; Ehime University Graduate School of Medicine, JAPAN

## Abstract

Muscle quality is well-known to decrease with aging and is a risk factor for metabolic abnormalities. However, there is a lack of information on race-associated differences in muscle quality and other neuromuscular features related to functional performance. This study aimed to compare muscle quality, function, and morphological characteristics in Japanese and Brazilian older individuals. Eighty-four participants aged 65–87 years were enrolled in the study (42 Japanese: 23 men, 19 women, mean age 70.4 years; 42 Brazilians: 23 men, 19 women, mean age 70.8 years). Echo intensity (EI) and muscle thickness (MT) of the quadriceps femoris were measured using B-mode ultrasonography. A stepwise multiple linear regression analysis with EI as a dependent variable revealed that MT was a significant variable for Japanese participants (R^2^ = 0.424, P = 0.001), while MT and subcutaneous adipose tissue (SCAT) thickness were significant variables for Brazilian participants (R^2^ = 0.490, P = 0.001). A second stepwise multiple linear regression analysis was performed after excluding MT and SCAT thickness from the independent variables. Sex and age for Japanese participants (R^2^ = 0.381, P = 0.001) and lean body mass and body mass index for Brazilian participants (R^2^ = 0.385, P = 0.001) were identified as significant independent variables. The present results suggest that MT is closely correlated with muscle quality in Japanese and Brazilian older individuals. Increases in muscle size may induce decreases in intramuscular adipose tissue and/or connective tissues, which are beneficial for reducing the risks of metabolic impairments in Japanese and Brazilian older individuals.

## Introduction

Aging is an irreversible physiological phenomenon that causes functional impairments and morphological changes in skeletal muscle. In a cross-sectional study carried out by Lexell et al. [1], the reduction in cross-sectional area of the vastus lateralis (VL) muscle was up to 1% per year after 50 years of age. Meanwhile, in a 12-year longitudinal study by Frontera et al. [2], the quadriceps femoris (QF) cross-sectional area decreased by 10.3%, reflecting an annual reduction of 0.9%. These studies clearly showed that aging has a large impact on skeletal muscle quantity. More importantly, a cohort study involving a large population identified an association between age-related functional deterioration of skeletal muscle and mortality [3].

Muscle echo intensity (EI) has been widely employed for muscle quality evaluation using B-mode ultrasonography. EI is considered to provide important physiological information on the quantity of adipose tissue and/or connective tissue within skeletal muscle [4–9]. In particular, higher EI reflects lower muscle quality and was identified as a risk factor for metabolic abnormalities, which are associated with type 2 diabetes [10–13], and poor muscle functional capacity in older individuals.

While the effect of aging on muscle quality is well-documented, there is limited evidence on race-associated differences in muscle quality. Melvin et al. [14] compared EI and body composition between black people and white people, and showed that black people had lower EI and higher subcutaneous adipose tissue (SCAT) thickness than white people. To our knowledge, this was the first study to compare the effects of race on muscle EI. Other studies have focused solely on the influence of racial differences in adipose tissue, insulin resistance, or muscle size. Gallagher et al. [15] compared intermuscular adipose tissue (IMAT) and visceral adipose tissue among healthy sedentary African American, Asian, and white adults. They found greater amounts of IMAT in African American adults and smaller amounts of IMAT in Asian and white adults, suggesting the existence of race-associated differences in IMAT. Another study by Miljkovic et al. [16] compared IMAT of the calf muscle among 1623 Caucasian and Afro-Caribbean older women. They found that Afro-Caribbean women had significantly larger IMAT than Caucasian women with and without adjustment for other muscle and adipose tissue variables. Although these studies suggested the possible existence of race-related differences in muscle quality, there is a lack of studies involving comparisons among other races. To determine the criteria for sarcopenia from various points of view, such as muscle quality and muscle quantity, it would be valuable to achieve better understanding of differences according to race. Although Asian people are relatively thin, fat was shown to disproportionately accumulate in the abdominal region and may be a risk factor for increased insulin resistance [17]. There was also no difference in the relative amounts of IMAT to total adipose tissue among Asian and white men and women [17]. In the present study, we had the opportunity to compare muscle quality, functional capacity, and morphological characteristics between Japanese and Brazilian older individuals. Brazil is ranked 98th among 195 countries for body mass index (BMI) in men (25.5 kg/m^2^) and women (25.7 kg/m^2^) aged >18 years, while Japan is ranked 166th for BMI in men (22.6 kg/m^2^) and women (21.7 kg/m^2^) [18].

The purpose of this study was to compare muscle quality (EI), functional capacity, and morphological characteristics in Japanese and Brazilian older individuals. We hypothesized that Japanese participants would have lower EI, higher functional capacity, and better morphological characteristics compared with Brazilian participants.

## Materials and methods

### Study design and participants

The study had a cross-sectional design. Japanese participants were recruited through the Nagoya City Health Promotion Bureau from 2017 to 2019 and Brazilian participants were recruited using a database for participants involved in previous studies at the Exercise Research Laboratory (LAPEX), Federal University of Rio Grande do Sul from 2018 to 2019. All participants had no history of brain and heart disease, were able to do light–intensity exercise without problems, and had no anxiety about their lower back.

Forty-two Japanese older individuals (23 men: age, 69.3 ± 3.8 years; height, 166.0 ± 5.0 cm; weight, 63.4 ± 7.4 kg; BMI, 23.0 ± 2.3 kg/m^2^; 19 women: age, 71.7 ± 6.1 years; height, 152.3 ± 4.2 cm; weight, 50.4 ± 7.4 kg; BMI, 21.7 ± 3.0 kg/m^2^) and 42 Brazilian older individuals (23 men: age, 69.9 ± 5.1 years; height, 170.3 ± 6.2 cm; weight, 80.8 ± 11.1 kg; BMI, 27.8 ± 3.4 kg/m^2^; 19 women: age, 71.9 ± 6.8 years; height, 156.6 ± 6.6 cm; weight, 66.5 ± 13.9 kg; BMI, 26.9 ± 4.4 kg/m^2^) participated in the study. All participants in the Japanese group were Japanese. The participants in the Brazilian group comprised 30 white, 6 black, 5 multiracial, and 1 Japanese ancestry. The participants were healthy, habitually exercised for a few days per week, and were free of neurological, cardiovascular, and lower-extremity diseases. Before enrolment, all procedures, purposes, risks, and benefits associated with the study were explained and written consent was obtained. The study was approved by the Institutional Review Board of the Research Center of Health, Physical Fitness & Sports at Nagoya University (30–09) and the Federal University of Rio Grande do Sul (3.809.300), and was conducted in accordance with the guidelines of the Declaration of Helsinki.

### Experimental procedure

The Japanese participants visited the laboratory at Nagoya University on two different days separated by an interval of 2 weeks. On the first visit, they were interviewed about their physical conditions, daily life habits, and physical activity levels using questionnaires. On the second visit, demographic, functional, and morphological assessments were performed. The Brazilian participants visited the LAPEX at Federal University of Rio Grande do Sul on a single day. On that day, they were interviewed about their physical conditions, daily life habits, and physical activity levels using questionnaires before the assessments were performed. For both Japanese and Brazilian participants, body composition assessments and ultrasound measurements were initially performed after the participants had rested on a chair or bed for at least 15 min to avoid muscle contraction-induced fluid shifts during measurements. After completion of these measurements, functional capacity tests were performed. The order of the functional tests was random.

### Body composition measurements using bioelectrical-impedance analysis (BIA)

A BIA system (ITO-InBody370; ITO Co. Ltd., Tokyo, Japan) was used to measure body weight, %body fat, and lean body mass (LBM) for Japanese participants essentially as described previously [5]. Eight electrodes producing electric microcurrents of 20 kHz and 100 kHz were applied to the right and left hands and right and left feet. For accuracy of measurements, examiners ensured that the participants’ elbow and knee joints were fully extended and that the participants maintained a standing position with their legs and arms straight throughout the test. Height, weight, BMI, and waist circumference at the height of the umbilicus were also measured. Regarding test-retest reliability evaluated by the intraclass correlation coefficient (ICC), the ICC(2,1) values for body weight, body fat, LBM, BMI, and abdominal girth were 0.997, 0.987, 0.999, 0.985, and 0.992, respectively [5].

### Body composition measurements using dual energy X-ray absorptiometry (DXA)

A whole-body DXA machine (Lunar Prodigy; GE Healthcare, Madison, WI, USA) was used to measure body weight, %body fat, and LBM for Brazilian participants. The participants removed all metal objects and other items that could interfere with the scan, and were positioned in the center of the scanning table with their arms placed palm-down at their sides. Age, height, weight, sex, and ethnicity were entered into the computer, and the default software of the device was used to determine indices of body composition, including body weight, %body fat, and LBM. DXA is considered the gold standard methodology for measurement of body composition. Regarding precision errors, the coefficient of variation (CV) values were 0.82% for total fat mass and 0.86% for %body fat in 52 men and women aged 34.8 ± 8.4 (range, 20.1–50.5) years [19].

### Morphological measurement

**Fig 1:**
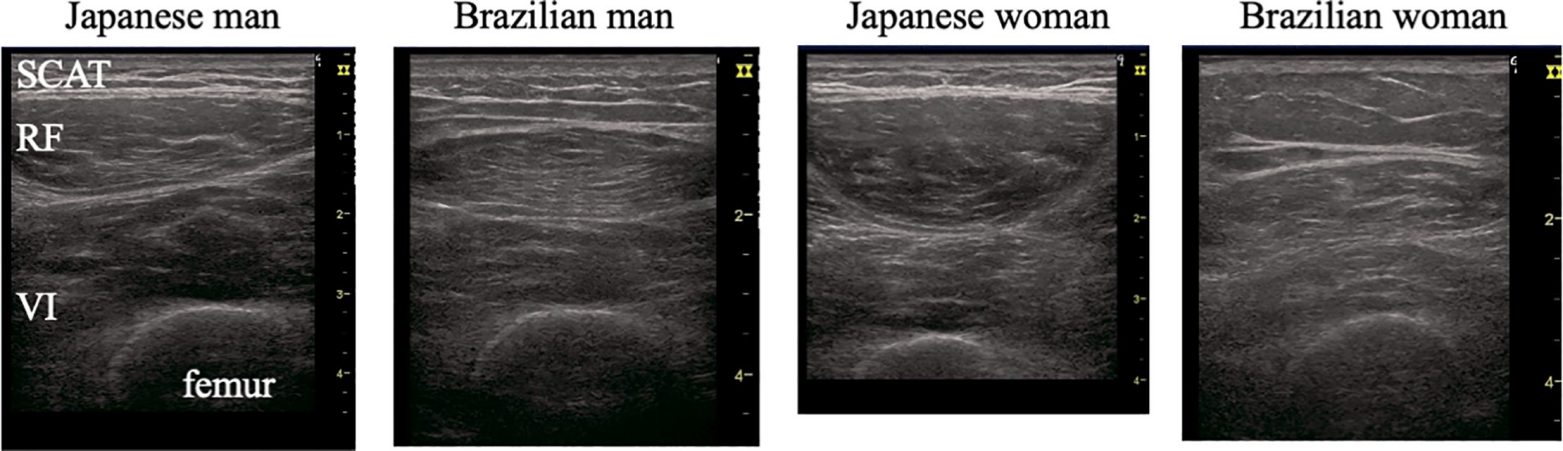
Representative B-mode ultrasound images at the anterior mid-thigh of Japanese and Brazilian older men and women. RF, rectus femoris; SCAT, subcutaneous adipose tissue; VI, vastus intermedius. The ultrasound images were taken with the internal RF tendon located at the center of each image.

Real-time B-mode ultrasonography (LOGIQ e; GE Healthcare, Chicago, IL, USA) with an 8–12-MHz linear-array probe (width, 3.8 cm) was used to measure the muscle thickness (MT), SCAT thickness, and EI at the anterior and lateral regions of the right mid-thigh essentially as described previously [9, 20, 21]. Fig 1 shows representative transverse ultrasound images of the anterior region of the QF in older Japanese and Brazilian men and women. All images were obtained by one researcher (A.Y.) with 7 years of experience in ultrasound measurements. For the anterior region, the SCAT thickness and MT of the rectus femoris (RF) and vastus intermedius (VI-ant) were measured, after ensuring that the internal RF tendon was placed at the center of each image (Fig 1). For the lateral region, the SCAT thickness and MT of the VL and VI (VI-lat) were measured at the midpoint between the greater trochanter and the lateral condyle of the tibia on the skin surface of the VL. Ultrasound images were obtained using the following acquisition parameters: frequency, 10 MHz; gain, 70 dB; depth, 4–8 cm; number of focal points, 1 (at top of image). The depth setting for the ultrasound images was changed depending on the MT of each participant. All measurements for the anterior and lateral mid-thigh were carried out in the supine position. The probe was coated with adequate water-soluble transmission gel to provide acoustic contact without depression of the dermal surface and was aligned perpendicular to the longitudinal axis of the QF to obtain transverse images. Three images were obtained for each measurement site. The ultrasound images were stored in an ultrasound device for future analysis.

The image files stored in the ultrasound device were subsequently transferred to a personal computer (iMac; Apple Inc., Cupertino, CA, USA) for storage. The SCAT thickness, MT, and EI of the target muscles were analyzed using ImageJ software (version 1.44; National Institutes of Health, Bethesda, MD, USA). MT of the RF and VL was defined as the distance between the superior border of the subcutaneous fascia and the deep aponeurosis. In the VI, MT was defined as the distance between the inferior border of the superficial aponeurosis and the superior border of the femur [5, 9].

The MT and SCAT thickness of the QF were calculated according to the following equations:

QFMT=(RFMT+VI‐antMT+VLMT+VI‐latMT)/4

QFSCATthickness=(anteriorSCATthickness+lateralSCATthickness)/2

The first step in the analysis of EI involved smoothing to convert the original grayscale ultrasound image to a 256-value grayscale image using the “smooth function” in ImageJ. The region of interest, which included as much muscle as possible but avoided visible bone and fascia, was determined using polygon selections to calculate the EI of each target muscle [20, 22]. The mean grayscale level within a region of interest was used as the EI, which was expressed in arbitrary units as a value between 0 and 255 (0 = black; 255 = white). The mean value of the EI in three images for each muscle was used for the analysis.

The EI of the QF was calculated using the following equation:

QFEI=(RFEI+VLEI)/2

### Functional measurements

The participants performed four functional tests in a gymnasium or laboratory (sit-up, supine-up, sit-to-stand, 5-m maximal walking) as described previously [5, 9, 23]. For the sit-up test, each participant lay supine with knees bent to approximately 90°, feet flat on the floor, and arms crossed on the chest. Each participant performed as many sit-ups as possible within 30 s with the examiner holding the participant’s ankles. The supine-up test timed each participant moving as quickly as possible from the supine position to standing, with no restriction on the manner of getting to the standing position. In the sit-to-stand test, participants were timed performing, as quickly as possible, 10 repetitions of standing from a sitting position with arms crossed on the chest. The 5-m maximal walk test consisted of taping four parallel lines on the floor at 1, 6, and 7 m (finish line) from the starting line (0 m). Subjects walked with maximal effort from the starting line toward the finish line, and the time it took to walk between the 1-m and 6-m lines was recorded by the examiner. The sit-up and sit-to-stand tests were conducted once. The supine-up and 5-m maximal walk tests were conducted twice, and the better result was used for further analysis.

### Physical activity level

The short version of the International Physical Activity Questionnaire (IPAQ) was used to obtain information on physical activity levels of the participants. We calculated and evaluated data on vigorous activity (days/week and min/day), moderate activity (days/week and min/day), and sedentary time (min/day) in this study.

### Statistics

All values are reported as mean and standard deviation. An unpaired-sample Student’s t-test was used to compare variables for demographic, functional, and morphological parameters between the two groups. Cohn’s *d* was calculated to evaluate effect size between the two groups. The Pearson product-moment correlation coefficient (r) was used to determine the association between variables. A partial correlation analysis was carried out with a control factor of SCAT thickness to estimate the effect of SCAT thickness on EI attenuation. A stepwise multiple regression analysis was performed by entering variables into a forward stepwise regression analysis if they made a significant contribution to the explained variance (put-in criteria, P ≤ 0.05; put-out criteria, P ≤ 0.100; as the default software settings) on two occasions. For the first step in the analysis, the dependent variable was EI of the QF and the independent variables were age, grip strength, sit-to-stand, MT of the QF, and SCAT thickness of the QF, with their selection based on our previous studies [4, 5] and simple correlation analyses. If any independent variables were identified as significant in the first step in the analysis, a second step in the analysis was performed with exclusion of the significant independent variables identified in the first step. To avoid multicollinearity in the analysis, we checked that the variance inflation factor was lower than the set criterion of 10 in all stepwise regression analyses. The level of significance was set at P < 0.05. All statistical analyses were performed using IBM SPSS Statistics for MacOS (version 26.0J; IBM Japan Corp., Tokyo, Japan).

## Results

Table 1 shows the demographic, functional, and morphological characteristics of the Japanese and Brazilian older individuals. There was no difference in mean age between Japanese (70.4 ± 5.1 years) and Brazilian (70.8 ± 5.9 years) participants (P = 0.211). However, Japanese participants were significantly shorter (Japanese: 159.8 ± 8.3 cm; Brazilians: 164.1 ± 9.4 cm; P = 0.026) and lighter (Japanese: 57.5 ± 9.8 kg; Brazilians: 74.3 ± 14.2 kg; P = 0.001) than Brazilian participants. Similarly, Japanese participants had smaller BMI (Japanese: 22.4 ± 2.7 kg/m^2^; Brazilians: 27.4 ± 2.9 kg/m^2^; P = 0.001), abdominal girth (Japanese: 85.2 ± 8.2 cm; Brazilians: 99.8 ± 11.7 cm; P = 0.001), %body fat (Japanese: 27.8 ± 7.3%; Brazilians: 37.7 ± 7.3%; P = 0.001), and LBM (Japanese: 35.2 ± 12.1 kg; Brazilians: 46.4 ± 10.5 kg; P = 0.001) than Brazilian participants.

**Table 1:** Demographic, functional, and morphological characteristics of Japanese and Brazilian older individuals.

	Japanese (N = 42)					Brazilians (N = 42)					
	Mean	±	SD		95% CI	Mean	±	SD	95% CI	Ratio of Japanese to Brazilians (%)	Effect size (d)
Demographic parameters											
Men/Women		23/19					23/19				
Age (years)	70.4	±	5.1		68.4, 71.6	70.8	±	5.9	69.0, 72.7	99.4	0.07
Height (cm)	159.8	±	8.3	*	157.8, 163.2	164.1	±	9.4	161.2, 167.1	97.4	0.48
Weight (kg)	57.5	±	9.8	†	55.1, 61.5	74.3	±	14.2	69.9, 78.8	77.4	1.38
BMI (kg/m2)	22.4	±	2.7	†	21.6, 23.4	27.4	±	3.9	26.2, 28.6	81.8	1.49
Abdominal girth (cm)	85.2	±	8.2	†	82.6, 88.3	99.8	±	11.7	96.2, 103.5	85.4	1.45
Body fat (%)	27.8	±	7.3	†	25.3, 30.3	37.7	±	7.3	35.4, 40.0	73.7	1.36
LBM (kg)	0.3	±	12.1	†	31.0, 39.7	46.4	±	10.5	43.1, 49.6	0.6	0.99
Physical activity											
Vigorous (day/week)	0.3	±	0.7	*	0.1, 0.8	1.1	±	1.7	0.6, 1.7	27.3	0.07
Vigorous (min/day)	20.5	±	37.5		3.8, 37.1	30.0	±	47.7	15.1, 44.9	68.3	0.22
Moderate (day/week)	1.2	±	1.6	**	0.4, 1.5	2.6	±	2.5	1.8, 3.4	46.2	0.83
Moderate (min/day)	47.2	±	51.4		18.0, 55.6	55.0	±	65.8	34.5, 75.5	85.8	0.33
Sedentary time (min/day)	300.0	±	147.2		221.3, 340.5	326.8	±	189.2	267.8, 385.7	91.8	0.28
Functional parameters											
Sit-up (reps)	10.8	±	6.7	**	8.2, 12.2	6.7	±	7.3	4.4, 8.9	161.2	0.59
Supine up (s)	2.9	±	0.8	†	2.6, 3.2	4.7	±	2.2	3.9, 5.3	61.7	1.09
Sit-to-stand (s)	11.9	±	2.5	†	11.3, 13.1	17.9	±	4.5	16.5, 19.3	66.5	1.65
5-m maximal walk (s)	2.3	±	0.3	**	2.2, 2.5	2.6	±	0.5	2.4, 2.8	88.5	0.73
Grip strength (kg)	28.5	±	7.5		25.9, 31.0	29.9	±	9.4	27.1, 32.9	95.3	0.16
Morphological parameters											
MT of RF (mm)	14.2	±	3.4		13.3, 15.6	14.8	±	4.6	13.3, 16.2	95.9	0.15
MT of VI-ant (mm)	13.4	±	4.4		12.3, 15.3	12.7	±	4.4	11.3, 14.1	105.5	0.16
MT of RF&VI-ant (mm)	27.6	±	6.9		25.9, 30.6	27.5	±	12.4	24.8, 30.1	100.4	0.01
MT of VL (mm)	17.0	±	3.8		16.2, 18.7	16.2	±	4.3	14.9, 17.5	104.9	0.20
VI-lateral MT (mm)	12.5	±	4.0		11.4, 14.1	13.5	±	4.3	12.1, 14.8	92.6	0.24
MT of VL&VI-lat (mm)	29.5	±	6.8		28.0, 32.5	29.7	±	7.5	27.4, 32.0	99.3	0.03
MT of QF (mm)	28.6	±	6.2		27.3, 31.3	28.6	±	7.5	26.2, 31.0	100.0	0.00
Anterior SCAT thickness (mm)	7.9	±	2.7	†	7.1, 9.0	12.4	±	5.3	10.7, 14.0	63.7	1.07
Lateral SCAT thickness (mm)	5.6	±	2.3	†	4.9, 6.6	9.2	±	4.5	7.8, 10.6	60.9	0.65
QF SCAT thickness (mm)	6.8	±	2.4	†	6.1, 7.8	10.8	±	4.8	9.3, 12.3	63.0	1.05

*, P < 0.05

**, P < 0.01

†, P < 0.001.

BMI, body mass index; CI, confidence interval; LBM, lean body mass; MT, muscle thickness; QF, quadriceps femoris; RF, rectus femoris; SCAT, subcutaneous adipose tissue; VI, vastus intermedius: VL, vastus lateralis

Japanese participants had significantly lower frequency of vigorous activity (Japanese: 0.3 ± 0.7 days/week; Brazilians: 1.1 ± 1.7 days/week; P = 0.05) and moderate activity (Japanese: 1.2 ± 1.6 days/week; Brazilians: 2.6 ± 2.5 days/week; P = 0.01) than Brazilian participants.

Overall, functional test performance was better in Japanese participants than in Brazilian participants. For example, there were significant differences between Japanese and Brazilian participants in the sit-up (Japanese: 10.8 ± 6.7 reps; Brazilians: 6.7 ± 7.3 reps; P = 0.001) and supine-up (Japanese: 2.9 ± 0.8 s; Brazilians: 4.7 ± 2.2 s; P = 0.001) tests. There was no significant difference in grip strength between the two groups (Japanese: 28.5 ± 7.5 kg; Brazilians: 29.9 ± 9.4 kg; P = 0.427).

There were no significant differences in any MT parameters between Japanese and Brazilian participants. Meanwhile, anterior SCAT thickness (Japanese: 7.9 ± 2.7 mm; Brazilians: 12.4 ± 5.3 mm; P = 0.001), lateral SCAT thickness (Japanese: 5.6 ± 2.3 mm; Brazilians: 9.2 ± 4.5 mm; P = 0.001), and QF SCAT thickness (Japanese: 6.8 ± 2.4 mm; Brazilians: 10.8 ± 4.8 mm; P = 0.001) were significantly lower in Japanese participants compared with Brazilian participants.

**Fig 2:**
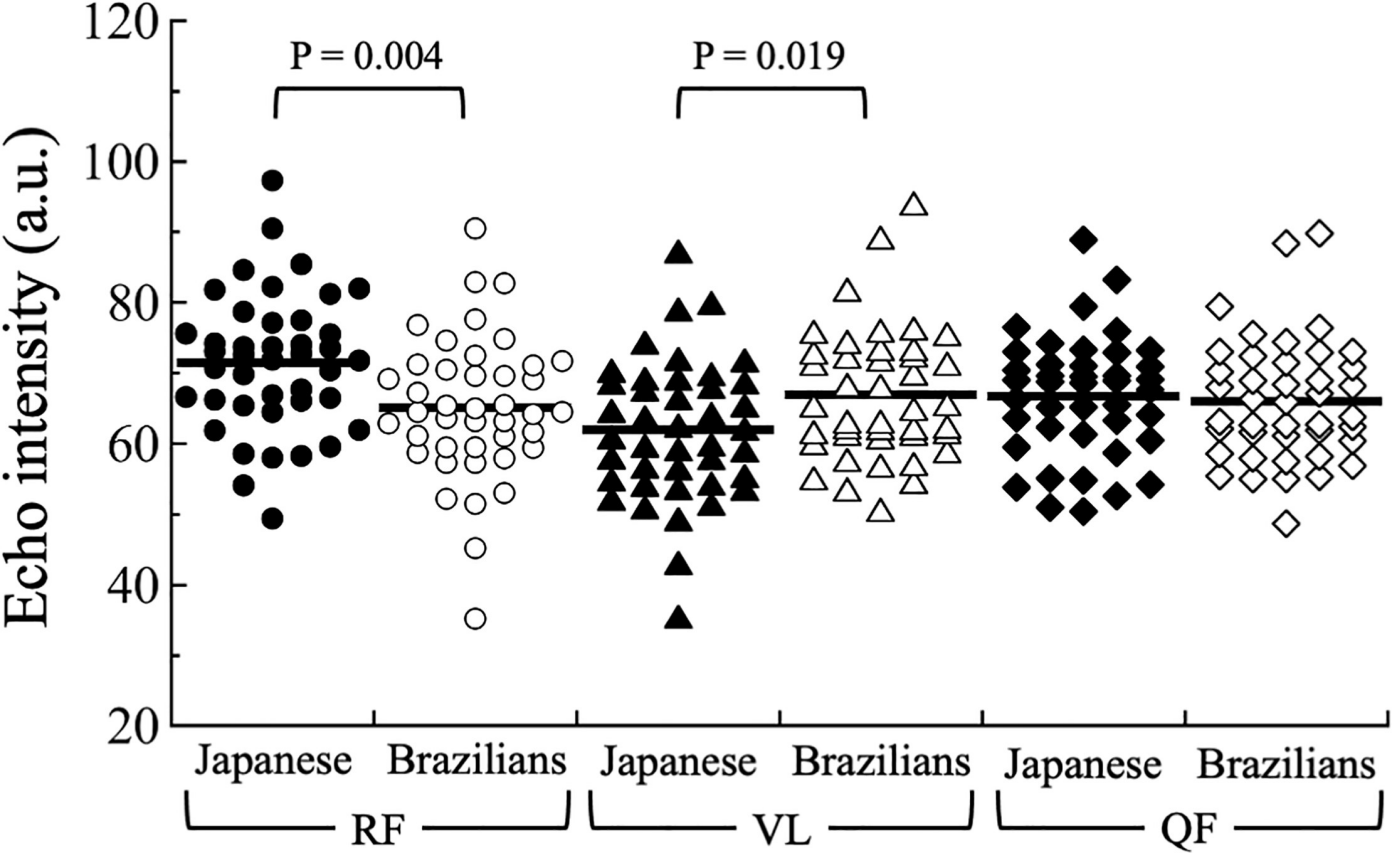
Comparison of the echo intensities of the RF, VL, and QF between Japanese and Brazilian participants. RF, rectus femoris; VL, vastus lateralis; QF, quadriceps femoris.

The EIs of the RF, VL, and QF in the participants are shown in Fig 2. The EIs of the RF and VL in Japanese participants were significantly higher and lower, respectively, than those in Brazilian participants (Japanese RF: 70.9 ± 9.5 a.u.; Brazilian RF: 65.1 ± 10.1 a.u.; P = 0.004; Japanese VL: 61.3 ± 9.6 a.u.; Brazilian VL: 66.9 ± 9.2 a.u.; P = 0.019). There was no significant difference in EI of the QF between Japanese and Brazilian participants (Japanese: 66.1 ± 8.1 a.u.; Brazilians: 66.0 ± 8.7 a.u.; P = 0.707).

Table 2 shows the relationships between the EIs of the RF, VL, and QF and the demographic and functional parameters in the participants. In Japanese participants, EIs of the RF, VL, or QF were significantly correlated with certain variables such as age (r = 0.348 to 0.438, P = 0.024 to 0.004) and BMI (VL: r = –0.367, P = 0.003; QF: r = –0.444, P = 0.007). In Brazilian participants, common demographic parameters were significantly correlated with EIs of the RF, VL, and QF: body weight (r = –0.531 to –0.606, all P = 0.001), BMI (r = –0.399 to –0.524, P = 0.009 to 0.001), abdominal girth (r = –0.354 to –0.410, P = 0.013 to 0.007), and LBM (r = –0.470 to –0.595, P = 0.001 to 0.002).

**Table 2:** Relationship between echo intensity and demographic and functional parameters.

	Echo intensity of RF				Echo intensity of VL				Echo intensity of QF			
	Japanese		Brazilians		Japanese		Brazilians		Japanese		Brazilians	
Demographic parameters												
Age	0.348	*	0.161		0.410	**	0.249		0.438	**	0.226	
Body weight	-0.282		-0.531	**	-0.539	**	-0.558	**	-0.473	**	-0.606	**
BMI	-0.192		-0.524	**	-0.444	**	-0.399	**	-0.367	*	-0.517	**
Abdominal girth	-0.084		-0.381	*	-0.183		-0.354	*	-0.154		-0.410	**
Body fat	0.165		0.072		0.063		0.271		0.130		0.187	
LBM	-0.407	**	-0.470	**	-0.177		-0.595	**	-0.334	*	-0.590	**
Functional parameters												
Sit-up	-0.124		-0.179		-0.117		-0.229		-0.139		-0.227	
Supine-up	-0.324	*	0.191		0.178		0.207		0.288		0.222	
Sit-to-stand	0.336	*	0.122		0.077		0.219		0.236		0.188	
5-m maximal walk	0.174		0.156		0.173		0.229		0.198		0.214	
Grip strength	-0.387	*	-0.239		-0.543		-0.458	**	-0.535	**	-0.383	*

*, P < 0.05

**, P < 0.01.

BMI, body mass index; LBM, lean body mass; QF, quadriceps femoris; RF, rectus femoris; VL, vastus lateralis

There were several significant relationships between EIs and functional parameters. In Japanese participants, EI of the RF was significantly correlated with supine-up (r = –0.324, P = 0.036), sit-to-stand (r = 0.336, P = 0.030), and grip strength (r = –0.387, P = 0.011), and EI of the QF was significantly correlated with grip strength (r = –0.535, P = 0.001). In Brazilian participants, EIs of the VL and QF were significantly related with grip strength (VL: r = –0.458, P = 0.002; QF: r = –0.383, P = 0.012).

To assess the effect of SCAT thickness on the relationships between EIs and MTs, we performed at partial correlation analysis with control factor of SCAT thickness for each measurement site. The partial correlation coefficients (Pearson’s r) between EIs and MTs of the RF, VL, and QF were –0.625 (–0.615), –0.563 (–0.564), and –0.650 (–0.651) (all P = 0.001) in Japanese participants, respectively, and –0.722 (–0.667), –0.734 (–0.718), and –0.698 (–0.651) (all P = 0.001) in Brazilian participants, respectively.

Table 3 shows the relationships between functional capacity and LBM or %body fat in the participants. In Japanese participants, supine-up (r = –0.475, P = 0.001), sit-to-stand (r = –0.475, P = 0.001), and 5-m maximal walk (r = –0.475, P = 0.001) were significantly inversely correlated with LBM, and sit-up (r = –0.377, P = 0.014) and supine-up (r = 0.562, P = 0.001) were significantly correlated with %body fat. In Brazilian participants, all functional tests were significantly correlated with LBM or %body fat (r = –0.720 to 0.624, P = 0.040 to 0.001).

**Table 3:** Relationship between functional capacity and lean body mass (LBM) and %body fat in Japanese and Brazilian older individuals.

	Japanese				Brazilians			
	LBM		Body fat		LBM		Body fat	
Sit-up (reps)	0.285		-0.377	*	0.319	*	-0.720	**
Supine up (s)	-0.475	**	0.562	**	-0.462	**	0.624	**
Sit-to-stand (s)	-0.480	**	0.030		-0.475	**	0.450	**
5-m maximal walk (s)	-0.492	**	0.199		-0.546	**	0.533	**

*, P < 0.05

**, P < 0.01.

Table 4 shows the relationships between EIs of the RF, VL, and QF and anterior, lateral, and total MTs and SCAT thicknesses in the participants. In both Japanese and Brazilian participants, EIs of the RF, VL, and QF were significantly negatively correlated with MTs of the anterior and lateral QF (r = –0.311 to –0.718, P = 0.05 to 0.001) at almost all regions. EIs of the RF, VL, and QF were also significantly negatively correlated with MT of the QF (r = –0.439 to –0.691, P = 0.001).

**Table 4:** Relationship between echo intensity and morphological parameters.

	Echo intensity of RF				Echo intensity of VL				Echo intensity of QF			
	Japanese		Brazilians		Japanese		Brazilians		Japanese		Brazilians	
Anterior QF												
MT of RF	-0.615	**	-0.667	**	-0.507	**	-0.473	**	-0.644	**	-0.641	**
MT of VI	-0.264		-0.473	**	-0.659	**	-0.469	**	-0.533	**	-0.525	**
MT of RF&VI	-0.464	**	-0.614	**	-0.658	**	-0.506	**	-0.646	**	-0.628	**
SCAT thickness	0.153		-0.224		0.167		0.004		0.183		-0.128	
Lateral QF												
MT of VL	-0.264		-0.415	**	-0.564	**	-0.718	**	-0.477	**	-0.625	**
MT of VI	-0.299		-0.455	**	-0.458	**	-0.311	*	-0.437	**	-0.431	**
MT of VL&VI	-0.324	*	-0.504	**	-0.585	**	-0.592	**	-0.524	**	-0.609	**
SCAT thickness	0.270		-0.091		0.165		0.092		0.249		-0.003	
Total												
MT of QF	-0.439	**	-0.591	**	-0.691	**	-0.575	**	-0.651	**	-0.651	**
SCAT thickness of QF	0.207		-0.166		0.169		0.047		0.216		-0.071	

*, P < 0.05

**, P < 0.01

MT, muscle thickness; QF, quadriceps femoris; RF, rectus femoris; SCAT, subcutaneous adipose tissue; VI, vastus intermedius: VL, vastus lateralis

A stepwise multiple linear regression analysis was performed with EI of the QF as the dependent variable, and age, grip strength, MT and SCAT thickness of the QF, and sit-to-stand as independent variables (Table 5). In the first step of the analysis, EI of the QF was explained by MT of the QF in Japanese participants (R = 0.651, adjusted R^2^ = 0.424) and by MT and SCAT thickness of the QF in Brazilian participants (R = 0.700, adjusted R^2^ = 0.490).

**Table 5:** Stepwise regression analysis as a dependent variable of echo intensity of the quadriceps femoris.

Group	Dependent variables	Independent variables	Regression coefficient	SE	Standard regression coefficients	P	R	Adjusted R2
	Step 1							
Japanese	QF EI	QF MT	-0.904	0.167	-0.651	0.001	0.651	0.424
Brazilians	QF EI	QF MT	-0.831	0.137	-0.723	0.001	0.700	0.490
		QF SCAT thickness	-0.480	0.214	-0.266	0.031		
	Step 2							
Japanese	QF EI	Sex	7.614	2.201	0.449	0.001	0.618	0.381
		Age	0.556	0.219	0.330	0.015		
Brazilians	QF EI	LBM	-0.367	0.117	-0.443	0.003	0.644	0.415
		BMI	-0.670	0.318	-0.297	0.042		

BMI, body mass index; EI, echo intensity; LBM, lean body mass; MT, muscle thickness; SCAT, subcutaneous adipose tissue; SE, standard error; VI, vastus intermedius: VL, vastus lateralis; QF, quadriceps femoris.

Independent variables for Step1: age, grip strength, QF MT, QF SCAT, sit-to-stand.

Independent variables for Step 2: age, BMI, LBM, sex, sit-to-stand.

The second step of the stepwise multiple linear regression analysis was performed with EI of the QF as a dependent variable and exclusion of independent variables that showed statistical significance in the first step of the analysis (MT and SCAT thickness of the QF). In the second step of the analysis, EI of the QF was explained by sex and age in Japanese participants (R = 0.618, adjusted R^2^ = 0.381) and by LBM and BMI in Brazilian participants (R = 0.644, adjusted R^2^ = 0.415).

## Discussion

The purpose of this study was to compare muscle quality and functional capacity in Japanese and Brazilian older individuals. Overall, Japanese participants were smaller and lighter and had lower %body fat and smaller LBM than Brazilian participants. In addition, Japanese participants had better functional capacity such as sit up, sit-to-stand, and 5-m maximal walk than Brazilian participants. Although Brazilian participants had significantly larger LBM, Japanese participants had similar MT of the QF to Brazilian participants. The EI of the QF was explained by MT of the QF for both Japanese and Brazilian participants in the first step of the stepwise regression analysis. In the second step, different independent variables, i.e. sex and age for Japanese participants and LBM and BMI for Brazilian participants, were extracted to explain the EI of the QF.

Only limited data have been available regarding muscle quality in different races, particularly in Asian people. In the present study, Japanese participants had significantly higher functional capacity in four of five tests, i.e. sit-up, supine-up, sit-to-stand, and 5-m maximal walk, than Brazilian participants. When the correlation coefficients are closely examined, those for LBM were similar between Japanese and Brazilian participants, while those for body fat were higher in Brazilian participants than in Japanese participants (Table 3). These observations suggest that the difference in body fat between Japanese and Brazilian older individuals may have a greater effect on functional capacity than LBM. A recent study suggested that imaging-derived muscle quality based on magnetic resonance imaging indicators can help to identify older individuals at risk of developing sarcopenia at an early stage [24], and also provides helpful information for understanding the potential role of muscle quality in the present study.

The Brazilian participants had poor results for body composition and physical function. A reason for these findings may be the changes in physical characteristics of Japanese immigrants whose dietary habits have changed from Japanese to Brazilian style. Ferreira et al. [25] showed second-generation Japanese-Brazilians had significantly higher BMI, waist circumference, and fat intake in their diet compared with the first-generation immigrants, which may be explained by their lifestyle change from Japanese to Western diet. Regarding BMI, second-generation Japanese-Brazilians had mean BMI of 24.9 ± 3.6 kg/m^2^, which was higher than that of Japanese participants (22.4 ± 2.7 kg/m^2^) in the study. Furthermore, relative fat consumed in total energy intake was relatively higher in second-generation immigrants than the first-generation immigrants [25], suggesting that dietary patterns changed to a Western style in Brazil. Another important finding in the present study was that vigorous and moderate physical activity (days/week) was significantly lower in Japanese participants than in Brazilian participants; however, this difference did not appear to be the cause of the significant differences in physical characteristics such as BMI, abdominal girth, and body fat.

Interestingly, there were no significant difference in MTs of any regions between the two groups, even though Japanese participants had significantly smaller LBM than Brazilian participants. This result implies that relative muscle mass to the thigh was larger in Japanese participants than in Brazilian participants. Similar results were reported by Visser et al. [26], who demonstrated race-associated differences in thigh muscle size and leg performance in white and black older individuals with no significant differences in physical characteristics (height, weight, and BMI). This type of muscle mass distribution pattern in Brazilian participants would also have affected their functional performance in tests such as sit-to-stand and 5-m maximal walk.

Contrary to our expectations, the EI values did not satisfactorily explain the functional capacity in Japanese and Brazilian participants (Table 2). These results were inconsistent with previous studies. Akima et al. [5] showed a significant relationship between EI of the QF and sit-to-stand test in Japanese older men (r = 0.492, P < 0.05) and women (r = 0.385, P < 0.05). Lopez et al. [27] reported a significant relationship between EI of the QF and 30-s sit-to-stand test (r = –0.564, P < 0.001) in Brazilian sedentary older men. Rech et al. [8] reported similar results to Lopez et al. [27] in Brazilian older women (r = –0.493, P < 0.01). The reason for the conflicting results is unknown. One possible explanation is the likely existence of a sex-dependent specialty in functional tests [5]. In the present study, we analyzed men and women together, and this may have affected the relationships. In contrast, grip strength was a good index to explain EI in both Japanese participants (r = –0.535, P < 0.01) and Brazilian participants (r = –0.383, P < 0.05). Similarly, Fukumoto et al. [28] reported a significant negative correlation between EI of the QF and maximum isometric voluntary contraction of the knee extensors in 92 middle-aged and older individuals (r = –0.40, P < 0.01). Our results are consistent with the suggestion that age-related decreases in muscle strength may be associated with factors other than muscle size [5, 28].

The EIs of the RF, VL, and QF in the present study were inversely correlated with the MTs (Table 3), consistent with some previous studies [5, 29, 30]. Fukumoto et al. [28] also reported that EI of the QF was negatively correlated with MT of the QF (r = –0.33, P < 0.01). Our findings reinforce the suggestion that the loss of muscle contractile tissue with aging is larger than the decrease in muscle size. Further discussions regarding EI and MT are provided below.

We compared a Brazilian male participant who was a third-generation Japanese-Brazilian (66 years of age) and Japanese male participants. This makes it possible to investigate the effects of lifestyle-related differences on body composition and muscle quality. His height and weight were 163.0 cm and 65.7 kg, respectively, and thus his BMI was 24.7 kg/m^2^, which was slightly higher than the mean BMI in Japanese male participants (23.0 kg/m^2^). His grip strength (37.8 kg), MT (33.9 mm), and EI of the QF (62.1 a.u.) were similar to the mean values in Japanese male participants. Furthermore, he had hyperlipidemia and diabetes, but did not have hypertension, which would be a normal physical condition considering his age. Therefore, this Brazilian male participant with Japanese ancestry had a similar body profile to that of Japanese male participants.

In the stepwise regression analysis, MT of the QF was extracted to predict EI of the QF in both Japanese and Brazilian participants. The same relationship was found in a simple regression analysis in the present study (Table 4), and similar findings were demonstrated in previous studies on older individuals [5, 28] and stroke patients [30]. In support of this relationship, the EIs were significantly correlated with LBM in both Japanese and Brazilian participants, suggesting that muscle size is a strong predictor of EI in older individuals.

According to a study based on spin-spin relaxation time (T2) using magnetic resonance imaging, there was a significant positive relationship between T2 and age in the gastrocnemius muscle of humans [31], reflecting longer T2 with aging muscle. T2 is known to become longer with aging [31, 32] or inactivity such as bed rest [33]. The age-associated increase in T2 is also related to enlargement of the extracellular space with aging, which may arise through atrophy of Type II fibers [31, 34]. In our previous study, the EIs of the VL and biceps femoris (BF) were both moderately associated with extramyocellular lipids (VL: r = 0.485, P < 0.01; BF: r = 0.648, P < 0.01) determined by ^1^H-magnetic resonance spectroscopy [20]. Importantly, no significant correlation was found between the EIs and intramyocellular lipids [20], suggesting EI determined by ultrasonography mainly reflects lipids outside of muscle cells. Extramyocellular lipids are expected to accumulate in the extracellular space, and this seems to be the main reason for the significant negative correlations between EIs and MTs in this study.

In Brazilian participants, SCAT thickness of the QF was extracted as another independent variable to explain EI in the first step of the stepwise regression analysis. This result was unexpected, because there were no significant correlations between EIs and SCAT thickness at any regions of the QF (Table 4). Although we cannot effectively explain this relationship based on the present observations, it is reasonable that EI and SCAT thickness tended to have a negative correlation (Table 4). One possible explanation is that body fat may act as a body overload mechanism in daily life and this may help to improve muscle quality, similar to the effect of strength training [7, 9].

In the second step of the stepwise regression analysis, two completely different independent variables were selected between Japanese and Brazilian participants. For Japanese participants, sex and age were extracted to explain the EIs. Age is known as an index that affects muscle quality [13, 35, 36]. It is reasonable that age was selected as an independent variable because the EIs were significantly correlated with age (Table 2). The other selected independent variable was sex. In our previous study, there was no difference in EI of the QF between older men and women [5]. Nevertheless, sex was selected to explain the EIs in Japanese participants. As we did not investigate the sex-related differences in EI, the exact reason for this finding is unclear. In our previous study [5], the correlation between EI and age seemed to be stronger in men (r = 0.588, P < 0.01) than in women (r = 0.369, P < 0.05). This effect may have had an influence on the results in the present study.

For Brazilian participants, LBM and BMI were selected as independent variables to explain the EIs. Rech et al. [8] reported that EI of the QF was significantly correlated with BMI (r = –0.472, P < 0.01) in 55 Brazilian older women aged 70.3 ± 6.2 years. LBM and BMI are indices of skeletal muscle mass and degree of obesity, respectively. These indices were expected to be alternative parameters for the independent variables selected in the first step, i.e. MT and SCAT thickness. These results clearly showed that the amounts of muscle (LBM) and adipose tissues (BMI or abdominal girth) had strong effects on EI of the QF in Brazilian participants (Table 2).

### Limitations of this study

There were a few limitations to this study. The first limitation is that two different devices were used to measure the body compositions of Japanese and Brazilian participants. This was an unavoidable situation because of the difficulties associated with bringing BIA or DXA systems to another country. The agreement between BIA and DXA findings was examined in many previous studies. For example, BIA and DXA were found to be interchangeable methodologies [37] and agreement was confirmed between these methods for measurements of fat free mass in the arm and leg segments, despite overestimation of body composition in the trunk region [38]. Nevertheless, we were unable to exclude the possibility that the measurements for body composition in the present study may have included errors arising from the different devices used. However, these errors, if any, did not seem to strongly affect the results of the study, because there were significantly large differences between the groups in weight, BMI, abdominal girth, and SCAT thickness. Thus, we do not expect the difference to have affected the comparisons between the Japanese and Brazilian participants.

The second limitation is that attenuation of EIs may have been induced due to differences in SCAT thickness, particularly in Brazilian participants. SCAT thickness was significantly lower in Japanese participants compared with Brazilian participants and this could have affected the EIs. However, as shown in the results, the Pearson’s r values and partial correlation coefficients controlled by SCAT thickness were sufficiently small that they could be ignored for both groups. We concluded that the effect of the SCAT thickness difference between the groups on the EIs was negligibly small.

Finally, the results for the EIs of the RF and VL were not consistent between the Japanese and Brazilian participants. This inconsistency is unlikely to arise from the effects of the SCAT thickness difference between the races. In our previous study on Japanese older men and women, the EIs of the RF and VL were approximately 70 a.u. and 60 a.u., respectively [5, 9], and consistent with the present study. For Brazilian participants, the EIs of the RF and VL were similar (RF: 78.3 ± 12.5 a.u.; VL: 74.9 ± 14.8 a.u.) [39]. Thus, the slight difference between EIs of the RF and VL may be associated with race-related differences.

## Conclusions

In conclusion, the results of the present study revealed that MT, sex, and age were factors that explained the EIs in Japanese older individuals, while MT, SCAT thickness, LBM, and BMI were factors that explained the EIs in Brazilian older individuals. For Japanese participants, MT was the only changeable factor according to lifestyle, while all four variables are changeable by lifestyle in Brazilian participants. For Brazilian older individuals, the amounts of muscle and adipose tissue would have strong effects on muscle quality. These results suggest that muscle size is the most important factor to maintain higher muscle quality in both Japanese and Brazilian older individuals.

## Supporting information

S1 Data (XLSX)
